# Supplementary material for: Preventive interventions for diabetic foot ulcer adopted in different healthcare settings: A scoping review protocol
Source: PLoS One. 2024 Oct 2;19(10):e0306486. doi: 10.1371/journal.pone.0306486 (PMC11446456; doi:10.1371/journal.pone.0306486)
Supplement: S1 Appendix — Source: Adapted from Araújo [27]. (DOCX) [file pone.0306486.s002.docx]

**Appendix I.** Review question and search strategy formulated for the MEDLINE/PubMed database.

| **Review question** | What is known about preventive interventions for diabetic foot ulcers adopted in different healthcare settings? | | |
| --- | --- | --- | --- |
|  | **P** | **C** | **C** |
| **Extraction** | Persons with diabetic foot ulcers | Preventive interventions for diabetic foot ulcers | Healthcare settings |
| **Conversion** | diabetic foot | prevention / primary prevention / primary medical care / prophylaxis / preventive medicine / preventive health care | - |
| **Grouping** | **diabetic foot**; diabetic feet; diabetic foot syndrome; diabetic foot ulcer; diabetic foot; | **prevention**; preventive; preventive care; disease prevention; wellness initiatives; proactive healthcare; health maintenance; preventative medicine; **preventive medicine**; **preventive health care**; health preservation; risk reduction; **primary prevention; primary medical care;** primary care; primary intervention; primary interventions; **prophylaxis**; prophylaxy; disease prevention; disease prophylaxis" OR "health protection; preventive medication; preventive therapy; preventive treatment; prophylactic institution; prophylactic management; prophylactic medication; prophylactic therapy; prophylactic treatment; preventive interventions; prophylactic measures; early detection strategies | **Identification through document reading** |
| **Building** | ("diabetic foot" OR "diabetic feet" OR "diabetic foot syndrome" OR "diabetic foot ulcer" OR "diabetic foot ulcers") | **(prevention OR** preventive **OR** "preventive care" **OR** "disease prevention" **OR** "wellness initiatives" **OR** "proactive healthcare" **OR** "health maintenance" **OR** "preventative medicine" **OR** "**preventive medicine**" **OR** "**preventive health care**" **OR** "health preservation" **OR** "risk reduction" **OR "primary prevention" OR "primary medical care" OR** "primary care" **OR** "primary intervention" **OR** "primary interventions" **OR prophylaxis OR** prophylaxy **OR** "disease prevention" **OR** "disease prophylaxis" **OR** "health protection" **OR** "preventive medication" **OR** "preventive therapy" **OR** "preventive treatment" **OR** "prophylactic institution" **OR** "prophylactic management" **OR** "prophylactic medication" **OR** "prophylactic therapy" **OR** "prophylactic treatment" **OR** "prophylaxis" **OR** "preventive interventions" **OR** "prophylactic measures" **OR** "early detection strategies") | **Identification through document reading** |
| **Usage** | **("diabetic foot" OR** "diabetic feet" **OR** "diabetic foot syndrome" **OR** "diabetic foot ulcer" **OR** "diabetic foot ulcers") **AND (prevention OR** preventive **OR** "preventive care" **OR** "disease prevention" **OR** "wellness initiatives" **OR** "proactive healthcare" **OR** "health maintenance" **OR** "preventative medicine" **OR** "**preventive medicine**" **OR** "**preventive health care**" **OR** "health preservation" **OR** "risk reduction" **OR "primary prevention" OR "primary medical care" OR** "primary care" **OR** "primary intervention" **OR** "primary interventions" **OR prophylaxis OR** prophylaxy **OR** "disease prevention" **OR** "disease prophylaxis" **OR** "health protection" **OR** "preventive medication" **OR** "preventive therapy" **OR** "preventive treatment" **OR** "prophylactic institution" **OR** "prophylactic management" **OR** "prophylactic medication" **OR** "prophylactic therapy" **OR** "prophylactic treatment" **OR** "prophylaxis" **OR** "preventive interventions" **OR** "prophylactic measures" **OR** "early detection strategies") | | |

**Source:** Adapted from Araújo [27]
