# Supplementary material for: Preventive interventions for diabetic foot ulcer adopted in different healthcare settings: A scoping review protocol
Source: PLoS One. 2024 Oct 2;19(10):e0306486. doi: 10.1371/journal.pone.0306486 (PMC11446456; doi:10.1371/journal.pone.0306486)
Supplement: S2 Appendix — Database and name of platform used: MEDLINE/PubMed. Search conducted: November, 2023. (DOCX) [file pone.0306486.s003.docx]

# **Appendix II**: Search strategy

Database and name of platform used: MEDLINE/PubMed

Search conducted: November, 2023.

| **Database** | **Query** | **Records retrieved** |
| --- | --- | --- |
| MEDLINE | (("diabetic foot"[Text Word] OR "diabetic feet"[Text Word] OR "diabetic foot syndrome"[Text Word] OR "diabetic foot ulcer"[Text Word] OR "diabetic foot ulcers"[Text Word]) AND (prevention[Text Word] OR preventive[Text Word] OR "preventive care"[Text Word] OR "disease prevention"[Text Word] OR "wellness initiatives"[Text Word] OR "proactive healthcare"[Text Word] OR "health maintenance"[Text Word] OR "preventative medicine"[Text Word] OR "preventive medicine"[Text Word] OR "health preservation"[Text Word] OR "risk reduction"[Text Word] OR "primary prevention"[Text Word] OR "primary medical care"[Text Word] OR "primary care"[Text Word] OR "primary intervention"[Text Word] OR "primary interventions"[Text Word] OR prophylaxis[Text Word] OR prophylaxy[Text Word] OR "disease prevention"[Text Word] OR "disease prophylaxis"[Text Word] OR "health protection"[Text Word] OR "preventive medication"[Text Word] OR "preventive therapy"[ Text Word] OR "preventive treatment"[Text Word] OR "prophylactic institution"[Text Word] OR "prophylactic management"[Text Word] OR "prophylactic medication"[Text Word] OR "prophylactic therapy"[Text Word] OR "prophylactic treatment"[Text Word] OR "prophylaxis"[Text Word] OR "preventive interventions"[Text Word] OR "prophylactic measures"[Text Word] OR "early detection strategies"[Text Word])) OR ((((diabetic foot[MeSH Terms]) AND (primary prevention[MeSH Terms])) OR (diabetic foot[MeSH Terms])) AND (Prevention and Control[MeSH Subheading])) | 2,791 |

*The search was conducted using controlled subject headings combined with specific terms.
