# Supplementary material for: Preventive interventions for diabetic foot ulcer adopted in different healthcare settings: A scoping review protocol
Source: PLoS One. 2024 Oct 2;19(10):e0306486. doi: 10.1371/journal.pone.0306486 (PMC11446456; doi:10.1371/journal.pone.0306486)
Supplement: S3 Appendix — DM: diabetes mellitus. * Other variables considered important may be extracted during the full-text reading. (DOCX) [file pone.0306486.s004.docx]

# **Appendix III**: Data extraction tool

| Study characteristics | | | | | | Participant characteristics | | | | | Intervention characteristics | | | | | Context |
| --- | --- | --- | --- | --- | --- | --- | --- | --- | --- | --- | --- | --- | --- | --- | --- | --- |
| ID | Author/Year | Country | Journal | Aim | Design | Sample | Sex | Age (mean) | Type of DM/Duration | Risk for ulceration | Type of intervention | Location | Duration | Professionals Involved | Main findings | Setting |
| 1 |  |  |  |  |  |  |  |  |  |  |  |  |  |  |  |  |
| 2 |  |  |  |  |  |  |  |  |  |  |  |  |  |  |  |  |
| 3 |  |  |  |  |  |  |  |  |  |  |  |  |  |  |  |  |

DM: diabetes mellitus

* Other variables considered important may be extracted during the full-text reading.
