# Supplementary material for: Preventive interventions for diabetic foot ulcer adopted in different healthcare settings: A scoping review protocol
Source: PLoS One. 2024 Oct 2;19(10):e0306486. doi: 10.1371/journal.pone.0306486 (PMC11446456; doi:10.1371/journal.pone.0306486)
Supplement: Attachment — Submitted filename: PONE-D-24-03451_reviewer.pdf [file pone.0306486.s005.pdf]

PLOS ONE

Preventive interventions for diabetic foot ulcer adopted in different healthcare settings: A scoping review protocol

--Manuscript Draft--

**Manuscript Number:**	PONE-D-24-03451
**Article Type:**	Study Protocol
**Full Title:**	Preventive interventions for diabetic foot ulcer adopted in different healthcare settings: A scoping review protocol
**Short Title:**	Diabetic foot ulcer prevention strategies
**Corresponding Author:**	Açucena Leal de Araújo, MSN<br>Universidade Estadual do Ceara - Campus do Itaperi: Universidade Estadual do Ceara<br>Fortaleza, Ceará BRAZIL
**Keywords:**	diabetes mellitus; preventive interventions; scoping review; diabetic foot ulcer
**Abstract:**	Background<br><br>Diabetic foot ulcers are challenging to heal, increase the risk of lower extremity amputation, and place a significant burden on patients, families, and healthcare systems. Prioritizing preventive interventions holds the promise of reducing patient suffering, lowering costs, and improving quality of life. This study describes a scoping review protocol that will be used to map preventive interventions for diabetic foot ulcers adopted in different healthcare settings.<br><br>Methods<br><br>The scoping review methodology was formulated in accordance with the PRISMA extension guidelines for scoping reviews and informed by the procedural insights provided by the JBI methodology group. Studies with participants diagnosed with type 1 and type 2 diabetes, aged 18 years or older, without an active ulcer at baseline, and studies of preventive interventions for foot ulcers in various healthcare settings will be screened. The search strategy was developed in collaboration with a research librarian using the PRESS checklist and no time or language limitations were applied. Data will be analyzed and summarized descriptively, including characteristics of studies, participants, and interventions.<br><br>Discussion<br><br>Understanding the strategies and gaps in diabetic foot ulcer prevention is critical. The literature can provide valuable insights for developing tailored interventions and strategies to effectively address these gaps, potentially accelerating progress toward improved outcomes in diabetic foot ulcer prevention.<br><br>Review registration<br><br>Open Science Framework DOI 10.17605/OSF.IO/FRZ97 [June 19, 2023]
**Order of Authors:**	Açucena Leal de Araújo, MSN<br>Francisca Diana da Silva Negreiros<br>Raquel Sampaio Florêncio<br>Thiago dos Santos Garces<br>Virna Ribeiro Feitosa Cestari<br>Samuel Miranda Mattos<br>Samara Jesus Sena Marques<br>Francisca Eridan Fonteles Albuquerque

Powered by Editorial Manager® and ProduXion Manager® from Aries Systems Corporation

	Wánderson Cássio Oliveira Araújo
	Vanessa de Brito Poveda
	Thereza Maria Magalhães Moreira
**Additional Information:**	
**Question**	**Response**
**Financial Disclosure**<br><br>Enter a financial disclosure statement that describes the sources of funding for the work included in this submission. Review the submission guidelines for detailed requirements. View published research articles from *PLOS ONE* for specific examples.<br><br>This statement is required for submission and **will appear in the published article** if the submission is accepted. Please make sure it is accurate.<br><br>**Funded studies**<br>Enter a statement with the following details:<br>• Initials of the authors who received each award<br>• Grant numbers awarded to each author<br>• The full name of each funder<br>• URL of each funder website<br>• Did the sponsors or funders play any role in the study design, data collection and analysis, decision to publish, or preparation of the manuscript?<br><br>Did you receive funding for this work?	The author(s) received no specific funding for this work.
**Competing Interests**<br><br>Use the instructions below to enter a competing interest statement for this submission. On behalf of all authors, disclose any competing interests that could be perceived to bias this work—acknowledging all financial support and any other relevant financial or non-financial competing interests.<br><br>This statement is required for submission	The authors have declared that no competing interests exist.

and **will appear in the published article** if the submission is accepted. Please make sure it is accurate and that any funding sources listed in your Funding Information later in the submission form are also declared in your Financial Disclosure statement.<br><br>View published research articles from *PLOS ONE* for specific examples.<br><br>**NO authors have competing interests**<br><br>Enter: *The authors have declared that no competing interests exist.*<br><br>**Authors with competing interests**<br><br>Enter competing interest details beginning with this statement:<br><br>*I have read the journal's policy and the authors of this manuscript have the following competing interests: [insert competing interests here]*<br><br>* typeset	
**Ethics Statement**<br><br>Enter an ethics statement for this submission. This statement is required if the study involved:<br><br>• Human participants<br>• Human specimens or tissue<br>• Vertebrate animals or cephalopods<br>• Vertebrate embryos or tissues<br>• Field research<br><br>Write "N/A" if the submission does not require an ethics statement.<br><br>General guidance is provided below. Consult the submission guidelines for detailed instructions. **Make sure that all information entered here is included in the Methods section of the manuscript.**	N/A

**Format for specific study types**<br><br>**Human Subject Research (involving human participants and/or tissue)**<br>• Give the name of the institutional review board or ethics committee that approved the study<br>• Include the approval number and/or a statement indicating approval of this research<br>• Indicate the form of consent obtained (written/oral) or the reason that consent was not obtained (e.g. the data were analyzed anonymously)<br><br>**Animal Research (involving vertebrate animals, embryos or tissues)**<br>• Provide the name of the Institutional Animal Care and Use Committee (IACUC) or other relevant ethics board that reviewed the study protocol, and indicate whether they approved this research or granted a formal waiver of ethical approval<br>• Include an approval number if one was obtained<br>• If the study involved *non-human primates*, add *additional details* about animal welfare and steps taken to ameliorate suffering<br>• If anesthesia, euthanasia, or any kind of animal sacrifice is part of the study, include briefly which substances and/or methods were applied<br><br>**Field Research**<br><br>Include the following details if this study involves the collection of plant, animal, or other materials from a natural setting:<br>• Field permit number<br>• Name of the institution or relevant body that granted permission	
**Data Availability**<br><br>Authors are required to make the data underlying their research findings fully available, without restriction.<br><br>If the manuscript reports pilot or preliminary results, the data underlying those results must be made available at the time of publication. A Data Availability	Yes - Results are reported and the data are fully available

Powered by Editorial Manager® and ProduXion Manager® from Aries Systems Corporation

Statement describing where the data can be found is required at submission. Your answers to this question constitute the Data Availability Statement and will be published in the article, if accepted. When legal or ethical requirements prohibit public data sharing, authors must indicate how researchers may obtain access to the data. See the PLOS Data Policy and FAQ for detailed information.<br><br>**Important:** Stating 'data available on request from the author' is not sufficient. If your data are only available upon request, select 'No' for the first question and explain your situation in the text box.<br><br>For protocols without pilot or preliminary data, authors are strongly encouraged to state how they plan to share research data from their study when it is completed or published.<br><br>If your study includes pilot data, will they be made fully available without restriction?	
**Describe where the data may be found in full sentences. If you are copying our sample text, replace any instances of XXX with the appropriate details.**<br><br>• If **no pilot data are reported** in the manuscript, please enter the following: *No datasets were generated or analysed during the current study. All relevant data from this study will be made available upon study completion.*<br>• If the manuscript reports pilot data **held in a public repository**, include URLs, accession numbers or DOIs. If this information will only be available after acceptance, indicate this by ticking the box below. For example: *All XXX files are available from the XXX database (accession number(s) XXX, XXX.).*<br>• If the manuscript reports pilot data contained **within the manuscript and/or**	No datasets were generated or analysed during the current study. All relevant data from this study will be made available upon study completion.

**Supporting Information files**, enter the following:<br>*All relevant data are within the manuscript and its Supporting Information files.*<br>• If neither of these applies but you are able to provide **details of access elsewhere**, with or without limitations, please do so. For example:<br><br>*Data cannot be shared publicly because of [XXX]. Data are available from the XXX Institutional Data Access / Ethics Committee (contact via XXX) for researchers who meet the criteria for access to confidential data.*<br><br>*The data underlying the results presented in the study are available from (include the name of the third party and contact information or URL).*<br>• This text is appropriate if the data are owned by a third party and authors do not have permission to share the data.<br><br>For study protocols with data management or sharing plans, authors are encouraged to briefly describe in their Data Availability Statement how data they generate will be made accessible when the study is completed. For example:<br>• Deidentified research data will be made publicly available when the study is completed and published.<br><br>* typeset	
Additional data availability information:	

Powered by Editorial Manager® and ProduXion Manager® from Aries Systems Corporation

Manuscript Click here to access/download;Manuscript;Manuscrito_PlosOne_2501.docx

Preventive interventions for diabetic foot ulcer adopted in different healthcare settings: A scoping review protocol

Short title: Diabetic foot ulcer prevention strategies

Açucena Leal de Araújo[1*], Francisca Diana da Silva Negreiros[2], Raquel Sampaio Florêncio[1], Thiago dos Santos Garces[3], Virna Ribeiro Feitosa Cestari[1], Samuel Miranda Mattos[3], Samara Jesus Sena Marques[1], Francisca Eridan Fonteles Albuquerque[4], Wánderson Cássio Oliveira Araújo[5], Vanessa de Brito Poveda[6,7], Thereza Maria Magalhães Moreira[1]

[1] Graduate Program in Clinical Care, Nursing and Health, Ceará State University, Fortaleza, Ceará, Brazil
[2] Walter Cantídio University Hospital, Federal University of Ceará, Fortaleza, Ceará, Brazil
[3] Graduate Program in Public Health, Ceará State University, Fortaleza, Ceará, Brazil
[4] Professional Master's Program in Health Management, University of São Paulo, São Paulo, Brazil
[5] Health Sciences Librarian, Federal University of Ceará, Fortaleza, Ceará, Brazil
[6] School of Nursing, University of São Paulo, São Paulo, Brazil
[7] Brazilian Center for Evidence-Based Health: JBI Center of Excellence, University of São Paulo, São Paulo, São Paulo, Brazil

* Corresponding author
E-mail: a.leal09@hotmail.com

Data availability statement: No datasets were generated or analysed during the current study. All relevant data from this study will be made available upon study completion.

Acknowledgments: We thank the Coordination for the Improvement of Higher Education Personnel (CAPES/Brazil) for the scholarships granted. We also thank the Graduate Program in Clinical Care, Nursing and Health at the Ceará State University, Brazil.

Funding: This study was partially funded by the Coordination for the Improvement of Higher Education Personnel – CAPES/Brazil - Financial Code 001.

Competing interests: The authors have declared that no competing interests exist.

Author contributions: Conceptualization: Açucena Araújo, Francisca Negreiros, Raquel Florêncio, Tsg, Samuel Mattos. Data curation: Wánderson Araújo, Açucena Araújo, Francisca Negreiros, Thiago Garces, Raquel Florêncio. Formal analysis: Açucena Araújo, Francisca Negreiros, Thiago Garces, Samuel Mattos, Samara Marques, Francisca Albuquerque. Investigation: Açucena Araújo, Francisca Negreiros, Raquel Florêncio, Thiago Garces, Samuel Mattos. Methodology: Açucena Araújo, Francisca Negreiros, Raquel Florêncio, Thiago Garces, Samuel Mattos. Validation and Visualization: Virna Cestari, Vanessa Poveda. Project administration and supervision: Açucena Araújo, Thereza Moreira. Writing – original draft, review & editing: All authors.

Abstract

Background: Diabetic foot ulcers are challenging to heal, increase the risk of lower extremity amputation, and place a significant burden on patients, families, and healthcare systems. Prioritizing preventive interventions holds the promise of reducing patient suffering, lowering costs, and improving quality of life. This study describes a scoping review protocol that will be used to map preventive interventions for diabetic foot ulcers adopted in different healthcare settings.

Methods: The scoping review methodology was formulated in accordance with the PRISMA extension guidelines for scoping reviews and informed by the procedural insights provided by the JBI methodology group. Studies with participants diagnosed with type 1 and type 2 diabetes, aged 18 years or older, without an active ulcer at baseline, and studies of preventive interventions for foot ulcers in various healthcare settings will be screened. The search strategy was developed in collaboration with a research librarian using the PRESS checklist and no time or language limitations were applied. Data will be analyzed and summarized descriptively, including characteristics of studies, participants, and interventions.

Discussion: Understanding the strategies and gaps in diabetic foot ulcer prevention is critical. The literature can provide valuable insights for developing tailored interventions and strategies to effectively address these gaps, potentially accelerating progress toward improved outcomes in diabetic foot ulcer prevention.

Review registration: Open Science Framework DOI 10.17605/OSF.IO/FRZ97 [June 19, 2023]

Introduction

Diabetes is one of the fastest growing global health emergencies of the 21st century. There are an estimated 537 million people with diabetes mellitus (DM) worldwide and 783 million by the year 2045 [1]. Lack of glycemic control, insulin resistance, microvascular and macrovascular dysfunction, among others, are possible complications [2]. Diabetic foot ulcers (DFUs) are the most common complication associated with neuropathy and/or peripheral arterial disease, causing high morbidity and mortality and significant financial costs to healthcare systems [3].

Diabetic peripheral neuropathy (DPN) reduces pain sensitivity, perception of plantar pressure, temperature and proprioception, and is present in half of all cases [4]. Symptoms of DPN can vary from person to person, but common complaints include numbness, tingling,

burning, paresthesia, hyperesthesia and pain that begins in the fingers and soles and progresses over months or years to involve the entire foot and ankle [5].

Pain is the most important natural mechanism that alerts people to seek medical attention. Thus, people with DM may not notice their injuries until they get worse [6]. These symptoms tend to worsen at night and at rest, as movement relieves the pain associated with nerve fiber degradation, as poorly myelinated sprouts appear that are responsible for stimuli that cause painful sensation [7]. In this context, DPN has been confirmed as a relevant risk factor for the development of DFU, deformity and lower extremity amputation [8].

At least one-fifth of people with DM will develop DFU at some point in their lives. Its annual incidence is 2-5% and its prevalence is 4-10% [9]. Recurrences occur in 40% of cases within one year and in 65% of cases within three years [10]. Lower extremity ulcers account for one-fifth of hospitalizations and a lower extremity is amputated every 30 seconds as a result of the disease [9]. Its complications are responsible for 40-70% of all non-traumatic lower extremity amputations worldwide [11].

A systematic review of economic evaluations found that DFU is a source of significant patient suffering, but also a significant cost to the individual and the healthcare system. The cost burden of DFU requires anywhere from 6 days to 5-7 years of patient income to cover the cost of treatment, with variation depending on the treatment setting and strategy. The annual cost of treating DFU is significantly higher than the cost of treating non-diabetic foot ulcers, estimated at US$1.38 billion versus US$0.13 billion. DFU infection places an additional burden on healthcare systems. The cost per admission for patients with and without DFU infection was significantly higher in the former group ($11,290 versus $8,145) [12].

The current approaches used by health services to prevent and treat these injuries at an early stage are many and varied. These include: i) identification of the foot at risk; ii) regular inspection and examination of the foot at risk; iii) education of patients, families and healthcare professionals about diabetes and PD; iv) ensuring routine use of appropriate footwear; and v) treatment of risk factors for ulceration [13]. These strategies help to minimize the incidence and recurrence of foot ulcers. However, efforts to prevent DFUs remain challenging and represent a significant global public health burden.

There are differences between countries in diabetes and foot care that should be considered when planning a scoping review. For example, people may have different risk factors for DFUs: peripheral neuropathy, foot deformities, history of ulcers, among others [14,15]. There may be differences in the care provided by the health systems of each country in the management of diabetes and its complications. A study on challenges and innovative

solutions to improve health outcomes in Latin America highlights the urgent need to prevent diabetes, but also complications related to the disease, such as DFUs, because of the burden they place on patients, societies, and health systems [16].

It is necessary to understand what preventive measures are being adopted globally, as this is essential for planning targeted health interventions and monitoring progress in health system performance in managing the prevention of DFUs over time. Therefore, a preliminary search for existing scoping reviews on this topic was conducted and no reviews were identified. The databases searched were Medical Literature Analysis and Retrieval System Online (MEDLINE) via Pubmed, JBI Evidence Synthesis and Open Science Framework.

Objective

The scoping review objective is to map the preventive interventions for diabetic foot ulcers used in different healthcare settings. Specifically, we aim to answer the following question: What is known about diabetic foot ulcer prevention interventions used in different healthcare settings?

Methods

A scoping review protocol was developed using the procedural insights provided by the JBI Scoping Review Methodology Group [17]. We will follow the steps suggested by Arksey and O'Malley [18], with additional recommendations from Peters et al. [17]: 1) define and align the objective and research question; 2) develop and align inclusion criteria with the objective and question; 3) describe the planned approach to evidence search, selection, data extraction, and presentation of evidence; 4) search for evidence; 5) select evidence; 6) extract evidence; 7) analyze evidence; 8) present results; and 9) summarize the evidence in relation to the purpose of the review, draw conclusions, and note any implications of the findings. The PRISMA extension for Scoping Reviews was used as the reporting framework [19].

The PPC (Population, Concept, Context) framework [17] was employed to formulate the research question. In this framework, P (population) represents individuals with either type 1 or type 2 diabetes mellitus, C (concept) pertains to preventive interventions for diabetic foot ulcers, and C (context) encompasses various healthcare scenarios.

Inclusion criteria

We will include studies conducted with participants diagnosed with type 1 and type 2 diabetes mellitus, aged 18 years or older, and without an active ulcer at the start of the study.

Pregnant women diagnosed with gestational diabetes will be excluded as hormonal changes occur during this period that may cause resistance to insulin action and are not a direct risk factor for DFU.

The main concepts of the study will include preventive interventions for diabetic foot ulcers related to foot self-management, footwear, structured education, foot-related exercises, orthotics, insoles [13], among others identified in the literature. Eligible studies must report outcome measures related to the use of interventions (preventive interventions that directly measure the presence or absence of DFU) (Chart 1).

Chart 1. Interventions for the prevention of diabetic foot ulcers.

**Interventions**	**Definitions**	**Examples**
Feet self management	Advanced assistive interventions (supportive technology) that the patient can use at home.	Home monitoring systems (infrared thermometer, thermal scales), lifestyle interventions, telemedicine/telehealth, technological applications, peer support programs.
Footwear	Broadly defined as any equipment for use on the feet, which may include insoles.	Prefabricated therapeutic footwear, custom-made therapeutic footwear.
Structured education	Any education modality offered in a structured manner. It can take various forms.	Educational programs, individual verbal education, motivational interviews, group educational sessions, video education, booklets, software, questionnaires, and pictorial education through cartoons or descriptive images.
Foot exercises	Any physical exercise specifically targeting the foot or lower limbs with the aim of altering foot physiology. These exercises are provided and/or supervised by a physiotherapist or healthcare professionals with appropriate training.	These exercises may include stretching and strengthening of the foot and ankle muscles and functional exercises such as balance and gait training.
Toe orthosis	Promotes alignment, pressure relief, spacing, and protection in the toes.	Custom-made silicone orthosis.
Insoles	May be a custom-made insole for the individual's foot using foot impression, mold, phenolic foam, and/or plaster, providing redistribution/relief of plantar pressure, cushioning, and postural alignment, or a flat or contoured	Custom-made insoles, prefabricated insoles.

	"ready-to-use" insole made without reference to the patient's foot shape.	

Source: Adapted from the Practical Guidelines of the International Working Group on the Diabetic Foot [13]

Regarding the context, we will include studies of preventive interventions used in different healthcare settings: primary, secondary, and tertiary healthcare facilities; home and community settings; and private clinics.

Types of sources

It will consider quantitative and qualitative primary studies that address issues related to interventions for the prevention of diabetic foot ulcers, without time or language limitations. Opinion articles, experience reports, ongoing trials, incomplete articles, book chapters, editorials and proceedings will be excluded.

Search strategy

The following databases will be searched: Scopus, Web of Science, MEDLINE via PubMed, Embase, Cummulative Index to Nursing and Allied Health Literature (CINAHL), The Cochrane Central Register of Controlled Trials (Cochrane Library), and Latin American and Caribbean Health Sciences Literature (LILACS) via Virtual Health Library (VHL).

To reduce publication bias and identify as much relevant evidence as possible, grey literature sources will also be consulted: Google Scholar, Brazilian Digital Library of Theses and Dissertations (BDTD in Portuguese), Catalog of Theses & Dissertations (CTD) of the Coordination for the Improvement of Higher Education Personnel (CAPES in Portuguese), Open Grey, and Open Access Theses and Dissertations (OATD). The first 100 records retrieved will be ranked by relevance.

Based on the established research question, we developed a search strategy (Appendix I) using the steps of the Extraction, Conversion, Combination and Use (ECCU) method proposed by Araújo [20], as shown in Chart 2. To ensure the feasibility of the method, we followed the Peer Checklist Review of Electronic Search Strategies (PRESS) guidelines [21].

Chart 2. Search strategy conducted on the MEDLINE/PubMed Database.

**Review question**	What is known about preventive interventions for diabetic foot ulcers adopted in different healthcare settings?		
	**P**	**C**	**C**
**Extraction**	Persons with diabetic foot ulcers	Preventive interventions for diabetic foot ulcers	Healthcare settings

**Conversion**	diabetic foot	prevention / primary prevention / primary medical care / prophylaxis / preventive medicine / preventive health care	-
**Grouping**	**diabetic foot**; diabetic feet; diabetic foot syndrome; diabetic foot ulcer; diabetic foot;	**prevention**; preventive; preventive care; disease prevention; wellness initiatives; proactive healthcare; health maintenance; preventative medicine; **preventive medicine**; **preventive health care**; health preservation; risk reduction; **primary prevention; primary medical care;** primary care; primary intervention; primary interventions; **prophylaxis**; prophylaxy; disease prevention; disease prophylaxis" OR "health protection; preventive medication; preventive therapy; preventive treatment; prophylactic institution; prophylactic management; prophylactic medication; prophylactic therapy; prophylactic treatment; preventive interventions; prophylactic measures; early detection strategies	**Identification through document reading**
**Building**	("diabetic foot" OR "diabetic feet" OR "diabetic foot syndrome" OR "diabetic foot ulcer" OR "diabetic foot ulcers")	**(prevention OR** preventive **OR** "preventive care" **OR** "disease prevention" **OR** "wellness initiatives" **OR** "proactive healthcare" **OR** "health maintenance" **OR** "preventative medicine" **OR** "**preventive medicine**" **OR** "**preventive health care**" **OR** "health preservation" **OR** "risk reduction" **OR "primary prevention" OR "primary medical care" OR** "primary care" **OR** "primary intervention" **OR** "primary interventions" **OR prophylaxis OR** prophylaxy **OR** "disease prevention" **OR** "disease prophylaxis" **OR** "health protection" **OR** "preventive medication" **OR** "preventive therapy" **OR** "preventive treatment" **OR** "prophylactic institution" **OR** "prophylactic management" **OR** "prophylactic medication" **OR** "prophylactic	**Identification through document reading**

		therapy" **OR** "prophylactic treatment" **OR** "prophylaxis" **OR** "preventive interventions" **OR** "prophylactic measures" **OR** "early detection strategies")	
**Usage**	(**"diabetic foot" OR** "diabetic feet" **OR** "diabetic foot syndrome" **OR** "diabetic foot ulcer" **OR** "diabetic foot ulcers") **AND (prevention OR** preventive **OR** "preventive care" **OR** "disease prevention" **OR** "wellness initiatives" **OR** "proactive healthcare" **OR** "health maintenance" **OR** "preventative medicine" **OR** "**preventive medicine**" **OR** "**preventive health care**" **OR** "health preservation" **OR** "risk reduction" **OR "primary prevention" OR "primary medical care" OR** "primary care" **OR** "primary intervention" **OR** "primary interventions" **OR prophylaxis OR** prophylaxy **OR** "disease prevention" **OR** "disease prophylaxis" **OR** "health protection" **OR** "preventive medication" **OR** "preventive therapy" **OR** "preventive treatment" **OR** "prophylactic institution" **OR** "prophylactic management" **OR** "prophylactic medication" **OR** "prophylactic therapy" **OR** "prophylactic treatment" **OR** "prophylaxis" **OR** "preventive interventions" **OR** "prophylactic measures" **OR** "early detection strategies")		

Source: Adapted from Araújo [20]

Four controlled vocabularies were used to develop the search strategy: Medical Subject Headings (MeSH), EMTREE from Embase, CINAHL Subject Headings, and Health Science Descriptors (DeCS in Portuguese). These controlled terms were combined with free natural language terms using the Boolean operators AND and OR to increase the sensitivity of the search and to obtain a broader range of relevant results [22].

To maintain an appropriate level of specificity, we chose to use terms related to the diabetic foot rather than specific terms related to diabetes mellitus and diabetic patients. Although the research question is focused on prevention and the population of interest is logically made up of people who have not yet developed diabetic foot, this logic would not directly apply to the search strategy. This is because the search strategy establishes a link between prevention measures and diabetic foot. If we included the broader terms (diabetes mellitus and diabetic patients), specificity would be compromised and work outside the scope of the research would be retrieved.

To broaden the scope of our search results, we interpreted the term associated with the concept including terms such as primary prevention and prophylaxis, which are directly related to disease prevention. To prevent the loss of valuable documents due to contextual limitations, we opted against explicitly defining terms from this thematic block in our search strategy. Instead, document selection will be based on thorough reading, as suggested by the authors, aiming to enhance qualitative control over the results obtained from bibliographic databases.

We will create a Portuguese variant for the LILACS bibliographic database. For the Scopus and Web of Science databases, which do not have an integrated controlled vocabulary, we will use the standard search strategy. For other bibliographic databases, we will use the standard search strategy with their respective subject headings.

All information sources, databases, and grey literature will be searched on the same day to mitigate potential bias. Initially, a preliminary search of the MEDLINE database via PubMed was conducted on November 28, 2023 (Chart 2), and it will subsequently be adapted for use with other electronic databases.

Ultimately, the literature search will conclude with a thorough review of the reference lists in the included documents, supplemented by insights and recommendations from experts in diabetes and diabetic foot ulcers.

Study/Source of evidence selection

The results obtained from information sources will be exported to the Rayyan QCRI Reference Manager [23]. This tool allows for fast and accurate processes, as it allows for the removal of duplicate studies, selection, and screening of studies. In addition, it maintains methodological rigor and transparency among reviewers, as it allows for blind review, thus avoiding potential bias [23].

Two investigators will independently review the titles and abstracts to identify potential articles of interest, and in the next step, the full text of preselected articles will be reviewed to confirm inclusion. A third investigator will resolve conflicts in the selection process.

The Cohen's kappa coefficient will be computed to assess agreement between reviewers during various selection phases, employing the following classification: 0 - 0.20 (none), 0.21 - 0.39 (minimal), 0.40 - 0.59 (weak), 0.60 - 0.79 (moderate), 0.80 - 0.90 (strong), and above 0.90 (almost perfect). If the agreement is 0.79 or less, training sessions will be organized among the raters to enhance the reliability of the process. To facilitate this, a subset of 30 studies will be selected for testing with the raters.

Studies that meet the inclusion criteria will proceed to the second phase. This includes reading the studies in full and selecting the studies that will be part of the review. Finally, the third stage involves a manual search of the references of the included studies. The entire identification, screening and inclusion process will be documented in the PRISMA flow chart.

Data extraction

Data extraction will be performed by two independent reviewers using a Microsoft Excel® spreadsheet. The accuracy of the information will be checked by the third reviewer. Disagreements and uncertainties will be discussed until a consensus is reached among all authors. Information mapping will be based on the adaptation of the JBI instrument to characterize scientific production [17].

The following data will be extracted from the studies: i) characteristics of the study (author, country of origin, year of publication, journal, objective(s), and study design); ii)

characteristics of the participants (sample size, sex, average age, risk of ulceration, and risk factors for ulceration); and iii) characteristics of the intervention (type of intervention, sample recruitment site, duration of intervention, professionals involved, and main outcomes of preventive interventions). Additional important variables will be extracted during the full-text reading process.

The draft version of the data extraction tool may be modified and revised as necessary during the data extraction process, with any adjustments documented in the scoping review report. Additionally, to address selective reporting within studies, a proactive approach will be taken to identify unpublished studies. Key researchers in the field will be contacted to inquire about any relevant unpublished data. Furthermore, in cases where data are incomplete or further clarification is needed, authors will be contacted directly to request missing or additional information.

Data analysis and presentation

The results will be analyzed and synthesized descriptively, employing a narrative synthesis approach. Initially, the studies will be grouped to offer a comprehensive overview of the scope, nature, and distribution of those included in the review, presented in a characterization table. Subsequently, the results will be discussed using thematic categories identified by the authors after thorough reading and exploration of the research encompassed in the review.

Discussion

This scoping review protocol endeavors to make a significant contribution to the field of diabetic foot ulcer prevention. The meticulous design of the search strategy ensures the comprehensive retrieval of relevant evidence. By consulting various databases and grey literature sources, utilizing controlled vocabularies, and applying Boolean operators, the protocol maximizes sensitivity in identifying pertinent studies. Through this comprehensive approach, the review aims to gather valuable evidence that can inform future research directions and enhance understanding of effective interventions for diabetic foot ulcers.

Expected outcomes of this thorough review process include the identification of research gaps and the synthesis of evidence to guide the development of tailored interventions. Ultimately, these contributions are anticipated to lead to improved patient outcomes and advance healthcare practices in the prevention of diabetic foot ulcers.

References

1. International Diabetes Federation. IDF Diabetes Atlas [Internet]. 10th edition. International Diabetes Federation; 2021 [cited 2023 Dec 12]. Available from: https://diabetesatlas.org/idfawp/resource-files/2021/07/IDF_Atlas_10th_Edition_2021.pdf
2. Dal Canto E, Ceriello A, Rydén L, Ferrini M, Hansen TB, Schnell O, et al. Diabetes as a cardiovascular risk factor: An overview of global trends of macro and microvascular complications. Eur J Prev Cardiol. 2019;26:25-32.
3. Netten JJ, Sacco ICN, Lavery LA, Monteiro-Soares M, Rasmussen A, Raspovic A, et al. Treatment of modifiable risk factors for foot ulceration in persons with diabetes: a systematic review. Diabetes Metab Res Rev. 2020;36(S1):e3271.
4. Edmonds M, Manu C, Vas P. The current burden of diabetic foot disease. J Clin Orthop Trauma. 2021;17:88-93.
5. Zakin E, Abrams R, Simpson DM. Diabetic Neuropathy. Semin Neurol. 2019;39(5):560-9.
6. Callaghan BC, Gallagher G, Fridman V, Feldman EL. Diabetic neuropathy: What does the future hold? Diabetology. 2020;63(5):891-7.
7. Peltier A, Goutman SA, Callaghan BC. Painful diabetic neuropathy. BMJ. 2014;348:g1799.
8. Lazzarini PA, Pacella RE, Armstrong DG, Van Netten JJ. Diabetes-related lower-extremity complications are a leading cause of the global burden of disability. Diabet Med. 2018.
9. Chun D, Kim S, Kim J, Yang HJ, Kim JH, Cho JH, et al. Epidemiology and burden of diabetic foot ulcer and peripheral arterial disease in Korea. J Clin Med 2019;8(5):748.
10. Armstrong DG, Boulton AJM, Bus SA. Diabetic foot ulcers and their recurrence. N Engl J Med 2017;376(24):2367-75.
11. Amoah VMK, Anokye R, Acheampong E, Dadson HR, Osei M, Nadutey A. The experiences of people with diabetes-related lower limb amputation at the Komfo Anokye Teaching Hospital (KATH) in Ghana. BMC Res Notes. 2018;11:66.
12. Woods TJ, Tesfay F, Speck P, Kaambwa B. Economic evaluations considering costs and outcomes of diabetic foot ulcer infections: A systematic review. PLoS One. 2020;15(4):e0232395.
13. International Working Group on the Diabetic Foot. IWGDF Guidelines on the prevention and management of diabetic foot disease [Internet]. International Working Group on the

Diabetic Foot; 2019 [cited 2023 Nov 15]. Available from: https://iwgdfguidelines.org/wp-content/uploads/2019/05/IWGDF-Guidelines-2019.pdf

14. Huang ZH, Li SQ, Kou Y, Huang L, Yu T, Hu A. Risk factors for the recurrence of diabetic foot ulcers among diabetic patients: a meta-analysis. Int Wound J. 2019 Dec 6;16(6):1373-82.
15. Yazdanpanah L, Shahbazian H, Nazari I, Arti HR, Ahmadi F, Mohammadianinejad SE, et al. Incidence and Risk Factors of Diabetic Foot Ulcer: A population-based diabetic foot cohort (ADFC Study)—two-year follow-up study. Int J Endocrinol. 2018;2018:7631659.
16. Avilés-Santa ML, Monroig-Rivera A, Soto-Soto A, Lindberg NM. Current state of diabetes mellitus prevalence, awareness, treatment, and control in Latin America: Challenges and innovative solutions to improve health outcomes across the continent. Curr Diabetes Rep. 2020;20(11):62.
17. Peters MDJ, Godfray C, McInerney P, Munn Z, Tricco AC, Khalil H. Chapter 11: Scoping reviews. In: Aromataris E, Munn Z, editors. JBI Manual for Evidence Synthesis [Internet]. Sydney: Joanna Briggs Institute; 2020 [cited 2023 Dec 10]. Available from: https://jbi-global-wiki.refined.site/space/MANUAL/4687342/Chapter+11%3A+Scoping+reviews.
18. Arksey H, O'Malley L. Scoping studies: Towards a methodological framework. Int J Soc Res Methodol. 2005;8(1):19-32.
19. Tricco AC, Lillie E, Zarin W, O'Brien KK, Colquhoun H, Levac D, et al. PRISMA Extension for Scoping Reviews (PRISMA-ScR): checklist and explanation. Ann Intern Med 2018;169(7):467-73.
20. Araújo WCO. Health information retrieval: Construction, models and strategies. Converg Cienc Inf. 2020;3(2):100-34. Portuguese.
21. McGowan J, Sampson M, Salzwedel DM, Cogo E, Foerster V, Lefebvre C. PRESS peer review of electronic search strategies: 2015 guideline statement. J Clin Epidemiol. 2016;75:40-6.
22. Siddaway AP, Wood AM, Hedges LV. How to do a systematic review: A best practice guide for conducting and reporting narrative reviews, meta-analyses, and meta-syntheses. Annu Rev Psychol. 2019:70:747-70.
23. Ouzzani M, Hammady H, Fedorowicz Z, Elmagarmid A. Rayyan—a web and mobile app for systematic reviews. Syst Rev. 2016;5(210).

Appendix I: Search strategy

MEDLINE – 2,791 records retrieved

Search conducted in November, 2023.

Database	Query	Records retrieved
MEDLINE	(("diabetic foot"[Text Word] OR "diabetic feet"[Text Word] OR "diabetic foot syndrome"[Text Word] OR "diabetic foot ulcer"[Text Word] OR "diabetic foot ulcers"[Text Word]) AND (prevention[Text Word] OR preventive[Text Word] OR "preventive care"[Text Word] OR "disease prevention"[Text Word] OR "wellness initiatives"[Text Word] OR "proactive healthcare"[Text Word] OR "health maintenance"[Text Word] OR "preventative medicine"[Text Word] OR "preventive medicine"[Text Word] OR "health preservation"[Text Word] OR "risk reduction"[Text Word] OR "primary prevention"[Text Word] OR "primary medical care"[Text Word] OR "primary care"[Text Word] OR "primary intervention"[Text Word] OR "primary interventions"[Text Word] OR prophylaxis[Text Word] OR prophylaxy[Text Word] OR "disease prevention"[Text Word] OR "disease prophylaxis"[Text Word] OR "health protection"[Text Word] OR "preventive medication"[Text Word] OR "preventive therapy"[Text Word] OR "preventive treatment"[Text Word] OR "prophylactic institution"[Text Word] OR "prophylactic management"[Text Word] OR "prophylactic medication"[Text Word] OR "prophylactic therapy"[Text Word] OR "prophylactic treatment"[Text Word] OR "prophylaxis"[Text Word] OR "preventive interventions"[Text Word] OR "prophylactic measures"[Text Word] OR "early detection strategies"[Text Word])) OR ((((diabetic foot[MeSH Terms]) AND (primary prevention[MeSH Terms])) OR (diabetic foot[MeSH Terms])) AND (Prevention and Control[MeSH Subheading]))	2,791

*The search was conducted using controlled subject headings combined with specific terms.

PRISMA-P checklist

Click here to access/download
Supporting Information
PRISMA-P-checklist.doc
