# Supplementary material for: Preventive interventions for diabetic foot ulcer adopted in different healthcare settings: A scoping review protocol
Source: PLoS One. 2024 Oct 2;19(10):e0306486. doi: 10.1371/journal.pone.0306486 (PMC11446456; doi:10.1371/journal.pone.0306486)
Supplement: Attachment — Submitted filename: Response to reviewers.docx [file pone.0306486.s007.docx]

Title: Preventive interventions for diabetic foot ulcer adopted in different healthcare settings: A scoping review protocol

ID: PONE-D-24-03451

**RESPONSE FOR REVIEWERS**

Dear Editor and Reviewers of Plos One,

We sincerely appreciate your insightful feedback on our manuscript. We have carefully considered each of your comments and suggestions and would like to address them accordingly. Please refer to the Table below.

| **Reviewer/editor comments** | **Author responses and revisions** |
| --- | --- |
| **Reviewer 1** |  |
| Background: The introduction provides a comprehensive overview of the differences in diabetes and foot care across countries, emphasizing the need to consider these variations in a scoping review. The mention of diverse risk factors for Diabetic Foot Ulcers (DFUs) and the impact of healthcare system differences adds depth to the understanding of regional disparities.  The statement rightly underscores the importance of recognizing and addressing variations in diabetes and foot care during a scoping review. However, specific examples of how risk factors for DFUs vary between countries or regions would enhance the clarity of the argument.  Exploring how healthcare system differences impact diabetes management, especially in foot care, would provide valuable insights. More elaboration on this aspect could strengthen the connection between the review and healthcare system contexts.  Further details on the findings of the study in Latin America, including challenges and innovative solutions, would be beneficial. Understanding the applicability of these insights to other regions could enhance the relevance of the scoping review.  Clarifying how DFUs contribute to the burden on patients, societies, and health systems, and providing examples of variations across countries, would add depth to the discussion.  Mentioning existing initiatives or strategies in certain countries to address the urgent need for preventing diabetes and its complications, as highlighted in the Latin American study, would contribute to a more comprehensive understanding.  The emphasis on understanding global preventive measures for DFUs is well-stated, highlighting the importance of planning targeted health interventions and monitoring progress in health system performance. | We have carefully considered your comments and made several revisions to the introduction section. We added examples of regional differences in DFU risk factors, elaborated on healthcare system impacts on diabetes management, and included citations for other studies that were not mentioned in the first version of the manuscript.  We also discussed burden of DFUs across Latin America and other countries. |
| Clarifying why a scoping review is necessary and detailing the expected contributions to new knowledge would strengthen this section. Further clarification on the specific information sought through the scoping review would be beneficial. | We have added a paragraph about the necessity of a scoping review and its expected contributions to new knowledge. |
| Objective:  The suggested clarification of the scoping review's aim, focusing on preventive interventions, patient consent, and contextual factors, is well-articulated and aligns with the broader goals outlined in the background. | Thank you for your suggestion. The objective has been revised to align with your suggestion. |
| Methodology:  Referencing the methodology paper on scoping reviews is relevant, providing a basis for best practice guidance. The inclusion of the full citation enhances the credibility of the review. | We appreciate the acknowledgment of referencing the methodology paper on scoping reviews. The full citation has been included to enhance credibility. |
| Chart 1:  The comment about the difficulty in understanding Chart 1 raises a valid concern. Additional clarification on how it differs from the objective or providing an example could improve comprehension. | Additional clarification on Chart 1 has been provided in the text that precedes it to address the difficulty in understanding. |
| Data Extraction:  Including an example of a data extraction sheet along with contextual information is an excellent suggestion. It would enhance transparency and help readers better understand the process. | The suggestion to include an example of a data extraction sheet along with contextual information has been implemented (see Appendix II: Data extraction tool). This addition enhances transparency and aids readers in understanding the process. |
| In summary, the review is well-structured and informative, with the suggested improvements aimed at enhancing clarity and depth in specific areas. | Thank you for your feedback! |
| **Reviewer 2** |  |
| It is a very well written manuscript, well referenced (except for reference#11), and adequate protocol for a scoping review. However, I find that the publication of a scoping review protocol does not contribute to scientific knowledge very much. This type of publication does not provide more information than what is already registered and made available to all (through OSF in this case). It feels as if the authors are duplicating publications from a single project which is borderline unethical. | While we respect the reviewer's perspective on scoping review protocols, we believe they provide valuable insights into study design and methodology. We assure the reviewer that our publication aims to contribute to scientific knowledge by synthesizing existing literature in a comprehensive manner. |
| **Reviewer 3** |  |
| This scoping review is interesting since it will map the preventive interventions available for diabetic foot ulcers adopted in different healthcare settings. | We appreciate the interest in our scoping review. We will emphasize the novelty of our review compared to existing ones by highlighting its focus on mapping preventive interventions for diabetic foot ulcers across various healthcare settings. |
| Introduction  Several systematic reviews (including a meta-analysis) related to the prevention of foot ulcers in patients with diabetes are available, such as:  - Prevention of foot ulcers in the at-risk patient with diabetes: a systematic review  - Prevention, assessment, diagnosis and management of diabetic foot based on clinical practice guidelines: A systematic review  - Preventing foot ulceration in diabetes: systematic review and meta-analyses of RCT data  Therefore, please elaborate more on the novelty of this scoping review compared to existing reviews. | In response to the request for elaboration on the novelty of our scoping review, we emphasized its unique focus on mapping preventive interventions for diabetic foot ulcers across diverse healthcare settings. This aspect was highlighted in the revised version of the manuscript. |
| Methods:  How will the authors conduct "critical appraisal" for the included studies? | Thank you for your inquiry on this matter. In our scoping review, we have opted not to conduct critical appraisal of included studies. Scoping reviews aim to comprehensively map existing literature regardless of quality, focusing on breadth rather than depth. Given our objective to identify preventive interventions for diabetic foot ulcers across healthcare settings, critical appraisal may not directly align with our aims. However, we will ensure transparency in reporting limitations associated with included studies. |
